# Comparative Analysis of Diagnostic Accuracy and Complication Rate of Transperineal Versus Transrectal Prostate Biopsy in Prostate Cancer Diagnosis

**DOI:** 10.3390/cancers17061006

**Published:** 2025-03-17

**Authors:** Salam Najjar, Cristian Mirvald, Alexandru Danilov, Apostolos Labanaris, Adrian George Vlaicu, Leonardo Giurca, Ioanel Sinescu, Cristian Surcel

**Affiliations:** 1 Nord Hospital, 010948 Bucharest, Romania; salam.najjar@drd.umfcd.ro; 2 Centre for Uronephrology and Renal Transplantation, Fundeni Clinical Institute, 022328 Bucharest, Romania; alexandru.danilov94@gmail.com (A.D.); ioanel.sinescu@umfcd.ro (I.S.);; 3 Faculty of General Medicine, Department of Urology, “Carol Davila” University of Medicine and Pharmacy, 050474 Bucharest, Romania; 4 Interbalkan Medical Centre, 555 35 Thessaloniki, Greece; labanaris@web.de; 5 Department of Urology, “CF2” Hospital, 011464 Bucharest, Romania; a.vlaicu2@gmail.com (A.G.V.); leonardo.giurca@drd.umfcd.ro (L.G.)

**Keywords:** transrectal prostate biopsy, transperineal prostate biopsy, cancer detection rate, TRUS complication rate, MRI-targeted prostate biopsy, prostate cancer, artificial intelligence, TRUS rebiopsy

## Abstract

Introduction: Transperineal prostate (TP) biopsy has emerged as a substantial alternative to the conventional transrectal (TR) approach for prostate sampling by its ability to sample specific areas of the prostate more effectively. The objective of this review is to conduct a comparative analysis of the current literature regarding diagnostic accuracy, complication rate and clinical outcome of transrectal vs. transperineal approaches in prostate biopsy-naïve patients and in repeated biopsy scenarios. Materials and Methods: An extensive search of the literature in PubMed, Scopus, and Web of Science was conducted between September 2010 and September 2024. We utilized a robust and comprehensive retrieval strategy including phrasing the two approaches as follows: (perineal or transperineal) and (rectal or transrectal). Conclusions: The transperineal and transrectal approaches show similar results in the detection of PCa in biopsy-naïve men, similar rates of infection, urinary retention and effectiveness managing biopsy-associated pain. However, in the rebiopsy scenario, the TP approach has demonstrated increased accuracy compared to the TR approach. This has significant implications in decision making and patient counselling.

## Simple Summary

This study compares two common methods of prostate biopsy: transperineal (TP) and transrectal (TR). Both techniques are effective in detecting prostate cancer, with similar overall accuracy. However, TP biopsies have advantages, especially in patients needing repeat biopsies, as they provide better sampling of certain areas of the prostate. Additionally, TP biopsies have a lower risk of infection since they do not pass through the rectum, reducing the chances of introducing bacteria into the body. However, they may cause more discomfort and require local or general anesthesia. TR biopsies, while more commonly used, have a slightly higher risk of complications, such as infections and rectal bleeding. The study suggests that TP biopsies could become more widely adopted due to their safety benefits, particularly as technology improves and costs decrease. Ultimately, the best biopsy method should be chosen based on a patient’s individual medical history and risk factors.

## 1. Introduction

Prostate cancer continues to be the most prevalent cause of cancer-related morbidity and mortality among males worldwide [1,2]. Early detection through prostate biopsies is essential for its effective management. The standard instrument for prostate cancer diagnosis is still a prostate biopsy (PBx). Benjamin Barringer performed the first documented attempt of a PBx in 1922 using a TP needle-punch biopsy technique. Despite being minimally invasive, the technique’s success rate was low; only 50% of biopsies properly retrieved prostate tissue [3]. In 1954, Kaufman carried out a needle biopsy by feeling for a suspected spot in a DRE and guiding a needle transperineally with a digit to take several tissue cores [4]. This technique was less invasive than an open perineal prostate biopsy, with a lesser risk of erectile dysfunction and rectal injury. Later, the development of a transrectal ultrasound-guided prostate biopsy (TRUS-TR PBx) with systematic sextant 12-core whole-gland sampling by Hodge in 1989 made the transrectal technique the most popular prostate biopsy method, offering a practical and effective way to sample prostate tissue [5].

The transperineal approach has gained in popularity due to its potential benefits, such as increased sensitivity in the anterior region of the prostate and decreased infection risk [6,7]. Hence, the European Urology Association’s prostate cancer guidelines increasingly recommend that the transperineal approach be considered the primary method of conducting a prostate biopsy. In 2003, Barzell and Whitmore developed a targeted prostate biopsy (TP-PBx) using a brachytherapy grid that divides the prostate into 24 zones. The grid reduces human error and ensures accurate, systematic prostate sampling for accurate biopsy localization [8].

The purpose of this review is to compare the detection rates of both approaches in terms of their diagnostic accuracy of prostate cancer in biopsy-naïve patients and in patients who require a repeat biopsy, and their complication rate.

## 2. Materials and Methods

**Figure 1:**
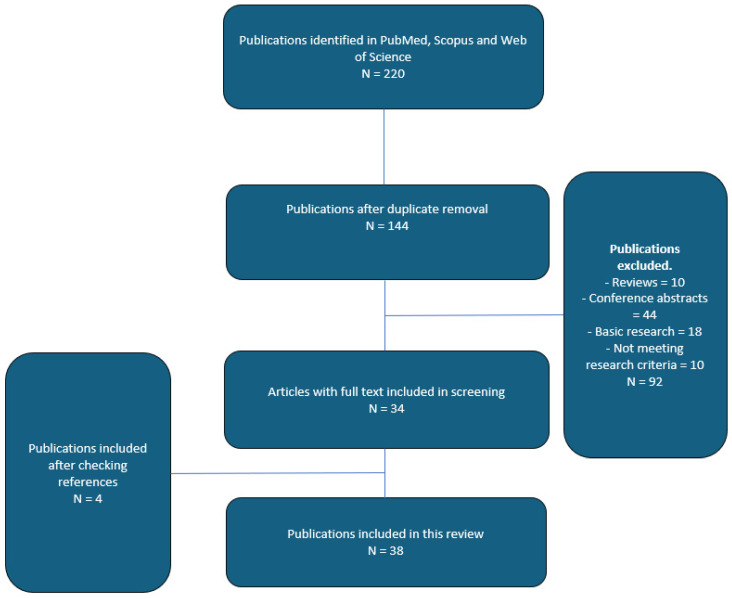
Flowchart of article selection process.

An extensive search of the literature in PubMed, Scopus, and Web of Science was conducted between September 2010 and September 2024. We utilized a robust and comprehensive retrieval strategy including phrasing the two approaches as follows: (perineal or transperineal) and (rectal or transrectal). Reference documents of the articles included in our study were also reviewed in their entirety to detect any other related studies. Our research parameters were the following: transrectal, transperineal, systematic, targeted biopsy, overall detection rates, clinically significant prostate cancer detection rates and complication rates in both biopsy-naïve and rebiopsy scenarios. We identified 220 potentially relevant publications. A total of 92 non-English studies were excluded due to language restrictions. Abstracts presented at different conferences and articles not available in full passages did not meet our selection criteria. The final number of publications included in this non-systematic review was 38 (see Figure 1).

## 3. Results

### 3.1. Diagnostic Accuracy in Biopsy-Naïve Patients in the PreMRI Era

Our review included 30 papers that compared the results of both techniques in terms of their diagnostic accuracy of prostate cancer (PCa). Overall prostate cancer detection rates varied between 25% and 56% with the transrectal (TR) approach and between 35% and 63% with the transperineal (TP) approach. In terms of clinically significant prostate cancer (csPCa), as defined by the EAU guidelines as ISUP grade group 3 and above [9], detection rates varied between 30.6% and 87% with the TR approach and between 36.9% and 84% with the TP approach. Mengxin Lu et al. [10] reported that the transperineal approach demonstrated a higher detection rate of anterior lesions, T1–T2 stages (30.6% vs. 36.9%) while the transrectal (TR) approach demonstrated a higher detection rate of csPCa, T3–T4 (72.4% vs. 62.5%). The higher detection rates in the TP biopsy in stages T1–T2 may be the result of better sampling of the anterior zone of the prostate, while in the TR biopsy, the needle has better access to the posterior and peripheral areas, with more biopsy cores passing through the prostate’s cancerous tissue [10]. One study showed that the transperineal biopsy had a greater cancer detection rate than the transrectal biopsy approach in patients with a PSA between 4 and 10 ng/mL [11], suggesting that patients with low- and intermediate-risk prostate cancer may benefit from a TP strategy. However, the detection rate of clinically significant prostate cancer was not significantly different between the TP group and the TR group (66.7% vs. 78.8%) [11]. In conclusion, our analysis shows that the overall prostate cancer detection and csPCa rates of both approaches are comparable (as seen in Table 1).

### 3.2. Diagnostic Accuracy in Biopsy-Naïve Patients in the MRI Era

Diagnostic Accuracy: A recent systematic review reports an increased cancer detection rate with an MRI-fusion biopsy, particularly in patients with prior negative biopsies. An MRI-targeted biopsy detects clinically significant malignancies in 72–87% of men with a previous negative biopsy and lowers the detection of clinically insignificant prostate cancer [18]. A significant factor in the precision of targeted biopsies is the level of experience of the biopsy operator [19]. Performing a lesion-targeted biopsy could decrease the number of biopsy cores taken, thereby reducing complications without diminishing detection rates. In our study, we compared transrectal (TR) vs. transperineal (TP) MRI-targeted biopsies in prostate cancer diagnosis; overall, clinically significant prostate cancer (csPCa) detection rates were comparable, between 50% and 80%. Qiyou Wu et al. [20]. showed that transperineal-targeted biopsy (TP-MRI-TBx) had higher detection rates for both PCa and csPCa and detected more csPCa in the anterior region of the prostate. Other studies, as seen in Table 2, showed that TP-MRIs had a higher likelihood of detecting PCa in the apex, anterior zone, and PIRAS-4 lesions, and allowed for better PCa risk assessment. A recent systematic review of a randomized trial by Uleri et al. [21] found no statistical difference in the detection rate of csPCa in both groups; however, the TP approach was favoured for anterior lesions. Ploussard et al. [22]. reported a higher prostate cancer detection rate in the posterior region for the TR group (59% vs. 44.3%), whereas prostate cancer was more frequently detected in the anterior region for the TP group (40.6% vs. 26.5%, respectively). Moreover, the prostate cancer detection rate in the TP group for ISUP < 2 was higher (23% vs. 9.4%). The overall prostate cancer detection rates were comparable between the two groups [22].

### 3.3. Cancer Detection Rate of Repeat Biopsies

The cancer detection rate remains low in biopsy-naïve patients, up to 56%, as previously discussed. However, urologists face clinical difficulties when patients have a negative biopsy yet a persistent clinical suspicion of prostate cancer, either because of an elevated PSA level, a palpably abnormal prostate in a DRE or an equivocal histology. A considerable percentage of these patients go on to have additional, occasionally several, transrectal ultrasound-guided biopsies (TRUS-Bxs) [26]. Despite current usage of a modified sextant biopsy protocol that is laterally guided, there is still a notably significant false-negative rate [27]. These patients are offered a saturation biopsy, which has a high PCa detection rate and can be performed transrectally or transperineally [28,29,30]. The transperineal method is becoming more and more popular, even though both saturation re-biopsy procedures seem to have comparable cancer detection rates [31]. We have included papers from the past 10 years that compare the overall cancer detection rate between the TP and TR groups. In saturation biopsy setups, cancer detection rates were increased in the TP group vs. the TR group (38.4% vs. 20.5%, respectively), as shown by Sergey et al. [32]. One recent study by Borkowetz et al. demonstrated that a combination prostate biopsy (systematic +fusion TP biopsy) has a higher detection rate of PCa, particularly for csPCa. In patients presenting with maxPI-RADS2, maxPI-RADS3, maxPI-RADS4 and maxPI-RADS5, the detection rate of csPCa was 14% (11/81), 28% (70/248), 43% (110/257) and 74% (91/124), respectively [33]. Pepe et al. showed that mpMRI/TRUS transperineal (TP) cognitive targeted biopsies have a higher detection rate of csPCa (93.3% vs. 78.3) [34]. Overall cancer detection rates were comparable in all studies, between 20% and 40%; however, csPCa and PCa in the anterior region were higher with the TP approach. Variability in the results between the studies, as seen below in Table 3, of csPCa detection was due to different biopsy setups, as Sergey et al. did not use the mpMRI fusion setup as the others did. This lowers the likelihood of sampling in the anterior and transitional zones, as overall prostate cancer and clinically significant prostate cancer (csPCa) were higher with the transperineal approach [34].

### 3.4. Complication Rates

The transperineal approach has been associated with lower rates of complication, such as urinary tract infections and sepsis [35]. Severe sepsis rates following TR biopsy have been reported in population-based investigations to range from 0 to 3% [36]. In healthcare systems, the management of such infectious problems results in a substantial financial burden [37]. RCTs, such as PREVENT, ProBE-PC and PERFECT, compare the safety and efficacy of transrectal versus transperineal prostate biopsies [22,38,39]. No difference in non-infectious complications between the two approaches have been reported. Postprocedural infection, sepsis and urinary retention were comparable, as seen in table below. The TP group reported more pain and discomfort, but was usually resolved within 1 week. Patient selection, use of five alpha reductase inhibitors, various biopsy techniques, number of biopsies, needle sizes and pain scores (not reported in all RCTs), limit the comparisons that can be made between the two approaches. A study by Huang et al. demonstrated that overall complication rates were higher in the transrectal (TR) group, as well as the rate of postinfectious complication (sepsis was 0% with the TP approach vs. 6.4% with the TR approach). The hospitalization rate was 0% in the TP group vs. 7.4% in the TR group [13]. Chen et al. showed that complication rates were 11.2% in the TR group vs. 6.1% in the TP group and non-septic UTIs were lower in the TP group [12]. One paper by Guo et al. showed higher rates of mild rectal bleeding and mild pain in the TP group [16].

The complication rate between transrectal and transperineal prostate biopsies are shown in Table 4 below.

#### Bleeding Complications Under Continuous Anticoagulant and Antiplatelet Therapy

There is a considerable risk of morbidity when patients undergo a TRUS biopsy. While haemorrhagic complications are far more common (haematuria, 39 to 44%, rectal bleeding, 17 to 27% and hematospermia, 12 to 16%) and can complicate surgery, these issues are usually minor and resolve on their own [42]. Urologists are now more frequently encountering patients with multiple comorbidities, such as vascular disease, deep-vein thrombosis, coronary arterial disease requiring previous intervention with angioplasty and the placement of stents (DESs), atrial dysrhythmias and ischemic heart disease [43], resulting in increasing numbers of patients on chronic anticoagulation and antiplatelet therapy. Due to haemorrhagic complications, there have historically been reservations about performing TRUS biopsies on patients receiving anticoagulant or antiplatelet treatment [44]. Clinical practices regarding the cessation of anticoagulation or antiplatelet therapy prior to TRUS biopsy vary greatly, with 95% of radiologists and 84% of urologists stopping anticoagulation (warfarin) treatment prior to TRUS biopsy and 52% of radiologists and 27% of urologists stopping antiplatelet (aspirin) therapy [45]. Saito et al. [46] assessed whether haemorrhagic complications associated with TP biopsies increased in patients on antiplatelet and/or anticoagulant therapy. TP biopsies were performed on 598 consecutive patients, 149 in the medication group and 449 in the control group. A modified Clavien classification system was used to compare and classify complications that developed in both groups. No anti-thromboembolic agent was stopped before, during, or after prostate biopsy in the medication group. Subgroup analyses were also conducted to predict bleeding risk using single antiplatelet, single anticoagulant and dual antiplatelet and/or anticoagulant medication. Haematuria (Grade I) developed in 88 (59.1%) and 236 (52.5%) patients in the medication and control groups, respectively. Clot retention (Grade I) was more frequent in the medication group (2.0% versus 0.2%). Neither group experienced complications of Grade III or higher. Haematuria was more frequent in patients taking a single anticoagulant (*p* = 0.007) or two anti-thromboembolic agents (*p* = 0.04) compared to those taking a single antiplatelet agent. Other complications were generally similar between the groups. In the multivariate analysis, the only significant risk factor for bleeding events was the use of more than two anti-thromboembolic medications.

Raheem et al. [47] assessed haemorrhagic complications regarding the continuation of anticoagulation/antiplatelet therapy during transrectal ultrasound-guided biopsy (TRUS). The biopsy setup was standardized to 12 cores. The anticoagulation/antiplatelet (group I) and control (group II) comprised 91 and 98 patients, respectively. Patients in group I subgroups were receiving low-molecular-weight heparin, warfarin, clopidogrel or aspirin either alone or in combination. Rectal haemorrhage was equally common in groups I (40%) and II (39%); haematuria was 46% in group I compared to 63% in group II; and haematospermia was 6% and 10% in groups I and II. Additionally, acute hospital admission due to clot retention was comparable at 2.2% in group I and 1% in group 2.

Choudhury et al. [48] assessed 902 patients in which warfarin and low-dose aspirin was not stopped prior to TRUS biopsy procedures. They demonstrated that chronic anticoagulation therapy was not associated with increased bleeding complications; however, there was an increased risk of minor bleeding complications with low-dose aspirin use. There were no sever bleeding complications. Additionally, the authors showed that an increased sampling number was associated with increased bleeding complications. They concluded that stopping warfarin and low-dose aspirin was usually not required and that performing a prostate biopsy with up to 10 cores is still a safe and acceptable procedure.

Due to various worldwide practices regarding the cessation of anticoagulant, bridge therapies and antiplatelet therapies, we cannot recommend the usage of a specific approach to decrease the risk of post-biopsy bleeding. The studies evaluated conclude that either TP or TR biopsy is feasible under continuous anticoagulant or antiplatelet treatment, with only a mild increase in the overall complication rate.

## 4. Discussion

Historically, a transrectal ultrasound has been the preferred procedure for a prostate biopsy, with a prostate cancer detection rate up to 40% [49]. Consequently, patients remain under suspicion of cancer even though they have undergone numerous procedures [50]. The primary advantage of a TP biopsy over the standard transrectal approach is enhanced sampling of the anterior and apical regions of the prostate, reduced risk of underestimation of PCa volume and grade and negligible rate of post-biopsy urosepsis [51]. It is also a beneficial alternative for patients who are unable or unwilling to undergo rectal procedures [52,53]. Accordingly, a TP biopsy is, in general, considered an alternative choice when a TR approach is unsuccessful or the number of collected samples is insufficient [54]. Although the TP approach presents some advantages over the TR approach, overall detection rates are comparable. Since both approaches are similar in terms of overall detection and csPCa detection rates, the urologist must tailor the approach according to the patient’s profile.

In patients with a negative biopsy, a TP approach is more accurate. According to a recent study, the TPBx group had a higher csPCa detection rate even though they had a much higher number of patients with a previous negative biopsy, which should lower the rate of biopsy positivity [55]. Also, TP demonstrates better accuracy in anterior and apical lesions. According to Zattoni et al. [23], TPBxs are substantially more likely than TRBxs to identify csPCa in the anterior zone (OR 5.62) and apex (OR 4.81). A meta-analysis that collected data from 3.522 and 5.140 patients who received TR and TP MRI-targeted biopsies, respectively, also demonstrated the benefit of detecting anterior (OR 2.17) and apical (OR 1.86) cancers [21].

The diagnostic accuracy of prostate cancer can be enhanced by identifying lesions that are suspicious for the disease and subsequently targeting them during biopsy using multiparametric magnetic resonance imaging [6].

Several technical improvements have been suggested to increase diagnostic accuracy. The application of cognitive TP biopsies was assessed in several papers. Valerio et al. compared software-assisted and visually directed targeted TP prostate biopsies, they discovered that the two approaches were nearly equal, with the software-based approach producing marginally better results (64% vs. 68% detection rate, respectively) [56]. Missed segments with TRUS biopsies were found anteriorly, 79%, in the anterior mid-prostate, 50%, and in the anterior apex, 23%. The most common segments involved in significant lesions missed with IB-MRI-TBx were dorsolateral, 58%, and apical, 37% [57]. The free-hand method for targeted TP biopsy has been demonstrated to be just as successful in detecting cancer as the TRUS-TR biopsy and can be carried out without the need of a brachytherapy grid [8].

The recent metanalysis compared direct in-bore guidance, MRI/ultrasound (US) fusion and cognitive guidance. No one technique was found to be clearly superior over the others [58]. In conclusion, both fusion and cognitive methods can improve csPCa detection rates, but these technologies are not available worldwide.

Although TP biopsies have demonstrated lower infection rates, this approach is not without risk: haematoma, local discomfort, transient urinary retention, etc. Serious complications such as bleeding and abscesses can occur, emphasising the importance of proper technique and patient selection. Similar to the ProBE-PC randomized trial, we did not find any significant difference between the two groups in terms of biopsy-related complications [11]. In the recent PREVENT trial, which randomized 658 patients to perform transperineal prostate biopsies without antibiotic prophylaxis versus transrectal prostate biopsies with targeted prophylaxis based on rectal cultures, found similar rates of infection and other overall complications. The PERFECT randomized trial revealed that there was no difference in grade ≥ 2 adverse events between TP (35.7%) and TR (40.5%, *p* = 0.4) biopsies, with only one TR patient developing grade 3 sepsis.

## 5. Limitations of the Study

The main limitation of our study is the non-systematic selection of papers included in our research. However, we tried to minimize this bias by including high-quality papers such as prospective and multicentric studies with a robust set of inclusion criteria. The majority of papers are monocentric, with various numbers of biopsies performed, which might have an impact on the interpretation of the data. Different biopsy procedures, needle sizes, MRI availability, inconsistent reporting of PSA prior to biopsy, cessation of anticoagulants and subgroup analysis for high-risk populations can also limit the generalisation of our conclusions.

Furthermore, there was inconsistent documentation on previous prostate treatment which could have influenced the results regarding PSA levels, prostate size and MRI reproducibility. Second, MRI readings and pathology analyses of specimens may be biased due to the absence of blinding to clinical features; also, the absence of a central review contributes to heterogeneity in MRI reporting and biopsy analysis. The lack of precise localization for index lesions may have affected results in subgroup analyses. Therefore, to assess the impact of these factors, multicentric prospective studies are urgently needed.

## 6. Cost-Effectiveness

For a long time, urologists have not widely performed TP biopsies due to the high cost of managing and preventing pain during the procedure. The primary disadvantages that increase the expense of a TP biopsy relative to a TR biopsy are the length of the process and the requirement for general anaesthesia. It has recently been demonstrated, meanwhile, that a TP biopsy is possible under local anaesthetic, with little discomfort and a mean operation time of 15.9 minutes [59]. A recent study shows that local anaesthetic transperineal biopsy is less expensive when compared to local anaesthesia transrectal biopsy and equally effective and cost-effective than general anaesthetic transperineal prostate biopsy. Since the majority of urologists are familiarized with the transrectal approach from residency, there is an innate resistance to switch to a TP approach. However, the TP approach is already performed in many countries, due to the advantage in better sampling and decreased postinfectious complications. With the increasing popularity of TP biopsies, the overall cost will decrease and it will become a cost-effective strategy [60].

## 7. Future Directions and Innovations

Emerging technologies, such as robotic assistance and refined imaging techniques, may further enhance the efficiency and effectiveness of the transperineal prostate biopsy [61,62]. Moreover, artificial intelligence (AI) has shown a lot of promise in interventional radiology; assistance with decision-making and outcome prediction has enhanced functionality. AI increased the performance of fusion imaging, robotics and software interactions [63]. Steiner et al. [64], showed that an AI-based assistive tool can improve pathologists’ evaluation of prostate biopsy samples in terms of precision, speed and consistency. In another study by Wang et al. [65], the prostate cancer detection rate was increased by an artificially intelligent ultrasound of the prostate (AIUSP), 49.5%, when compared to a transrectal ultrasound TRUS-guided 12-core systematic biopsy (34.60%) and mpMRI (35.80%) and the TRUS-SB (11.0%) and mpMRI (12.7%) groups had significantly lower total biopsy core positive rates than the AIUSP group (22.7%).

## 8. Conclusions

In terms of cancer detection rate, there are no significant differences between TR and TP systematic biopsies in biopsy-naïve patients. The transperineal technique showed higher cancer detection rates in anterior lesions, including patients with a history of negative transrectal biopsy. Transperineal prostate biopsy represents an important advancement in the field of urology and oncology. With its promising outcomes, reduced complication rate and improved patient comfort, it is poised to serve as a primary method of prostate sampling. Continued research and clinical trials will further clarify its role in prostate cancer diagnosis and management.

## Figures and Tables

**Table 1:** Diagnostic accuracy in biopsy-naïve patients in the preMRI era.

Author, Year	No. Pts	Study Type	Approach	Mean PSA	Overall CDR	csPCa Detection Rate	Key Findings
Liu, 2024 [11]	444	Retrospective	TR—228 TP—216	7	TR—22.4% TP—50%	TR—66.7% TP—78.8%	TPBx was associated with a greater CDR in grey zone PSA. csPCa detection rates did not significantly differ among systematic, targeted and combined biopsy groups.
Mengxin Lu, 2023 [10]	452	Retrospective	TR—245 TP—207	22	TR—56.3% TP—44.4%	TR—30.6% for T1 + T2 TP—36.9% for T1 + T2 TR—72.4% for T3 + T4 TP—62.5% for T3–T4	csPCa (T3–T4) was higher with TR approach, while detection rate of T1–T2 was higher in TP group.
Chen, 2021 [12]	212	Prospective	TR—86 TP—127	13.17	TR—50% TP—63.5%	TR—87.2% TP—84%	Overall CDRs and csPCa detection rates were comparable.
Huang, 2019 [13]	238	Comparative	TR—108 TP—130	10	TR—49% TP—45%	NA	CDRs were comparable.
K L Lo, 2019 [14]	200	Retrospective, comparative	TR—100 TP—100	>4	TR—25% TP—35%	TR—68% TP—50%	Overall CDRs were comparable.
Xue, 2017 [15]	4230	Meta-analysis	TR—1643 TP—1993	10	Comparable (OR = 1.11, 95%; CI = 0.92–1.34)	Comparable (OR = 0.76, 95%; CI = 0.61–0.96)	Overall CDRs and csPCa detection rates were comparable.
Guo, 2015 [16]	399	Prospective	TR—166 TP—173	9	TR—31.9% TP—35.3%	No difference	Overall CDRs and csPCa detection rates were comparable.
Cerruto, 2014 [17]	108	RCT	TR—54 TP—54	>4	TR—46.24% TP—44.44%	NA	Overall CDRs were comparable. TP approach offered better sampling of apex.

OR–odds ratio; CI—confidence interval; TP—transperineal; TR—transrectal; CDR—cancer detection rate; NA—not available; csPCa—clinically significant prostate cancer; TPBx—transperineal biopsy; pts—patients.

**Table 2:** Diagnostic accuracy in biopsy-naïve patients in the MRI era.

Author, Year	No. Pts	Study Type	Approach	Mean PSA	Overall Detection Rate	csPca Detection Rate	Key Findings
Qiyou Wu et al., 2024 [20]	8826	Retrospective	TR-MRI-TB TP-MRI-TB	NA	RR 0.91 [95% CI 0.76, 1.08]	RR 1.33 [95% CI 1.09, 1.63].	TP-MRI-TB had a higher detection rate of both PCa and csPCa. TP detected more csPCa in anterior region.
Fabio Zattoni, 2022 [23]	5241	Multicentric retrospective	TR-TBx—1936 TP-TBx—3305		TR-TBx—50% TP-TBx—64%	TR-TBx—35% TP-TBx—49%	TP-TBx had a significantly higher likelihood than TR-TBx of detecting csPCa in the apex, transition/central zone and anterior zone. It allowed for better PCa risk assessment.
Basil Kaufman, 2022 [24]	392	Comparative		7	Overall CDR—51%	Overall csPca—79%	CDR was comparable.
Alessandro Uleri, 2023 [21]	11 studies	Systematic and meta-analysis	TR—3522 TP—5140	NA	CDR—no statistical difference	(odds ratio [OR] 1.11, 95% [CI] 0.98–1.25; *p* = 0.1).	No statistical difference in detection of csPCa between TR and TP. In TP group, csPCa detection was higher in apical and anterior regions as well as in PI-RADS 4 lesions.
Romain Diamand, 2024 [25]	3949	Comparative	TP—2187 TR—1762	7	TR—45%. TP—51%	TR—45% for GG > 2 TP—51% for GG > 2 TR—23% for GG > 3 TP—29% for GG > 3	Higher CDR in anterior region and apex for TR.
Guillaume Ploussard, 2024 [22]	250	Randomized—PERFECT trial	TR—128 TP—122	7	TR-TBx—64.1% TP-TBx—71.3% sysTR—65.6% sysTP—60.2%	TR—51.9% for ISUP > 2 TP—46.4% for ISUP > 2	CDR in posterior region was higher in TR group, whereas CDR was higher in anterior region for TP group (40.6% vs. 26.5%). higher detection rate in the TP group for ISUP < 2 (23% vs. 9.4%). Overall CRD was comparable between two groups.

OR—odds ratio; RR—risc ratio; CI—confidence interval; TP—transperineal; TR—transrectal; CDR—cancer detection rate; NA—not available; PCa—prostate cancer; csPCa—clinically significant prostate cancer; sys—systematic; and TBx—targeted biopsy.

**Table 3:** Diagnostic accuracy of the transperineal vs. transrectal approach in a repeat biopsy setting.

Author, Year	No. Pts	Study Type	Approach	Mean PSA	Overall Detection Rate	csPCa Detection Rate	Key Findings
Chen, 2021 [12]	212	Prospective	TR—86 TP—127	13.17	TR—50% TP—63.5%	TR—87.2% TP—84%	CDR was comparable in both groups.
Borkowetz, 2019 [33]	710	Comparative	comP-bx (systemic +fusion)	8.8	Systematic cores = 12 Targeted cores = 7 CDR—40%	Overall csPCa—29–74%	fusPbx increased detection rate of PCa, particularly csPCa, than sysPbx alone.
Pepe, 2017 [34]	200	Comparative	TR—40 TP—56	NA	Overall CDR—30%	csPCa TR—78.3% TP—93.3%	mpMRI/TRUS TP cognitive targeted biopsies have higher detection rates of csPCa in anterior zone vs. mpMRI/TRUS TR fusion.
Kravchik, 2015 [32]	133	Comparative	STR—68 STP—65	7	No. of Cores TR—21 TP—45 CDR—comparable	TR—20.5% TP—38.4%	Template-guided approach had higher cancer detection rate of rebiopsy.

TP—transperineal; TR—transrectal; CDR—cancer detection rate; NA—not available; PCa—prostate cancer; csPCa—clinically significant prostate cancer; fusPbx—fusion biopsy; sysPbx—systematic biopsy; comP-bx—combination biopsy; TRUS—transrectal ultrasound; STR—saturated transrectal biopsy; and STP—saturated transperineal biopsy, mpMRI—multiparametric MRI.

**Table 4:** Complication rates between transrectal and transperineal prostate biopsies.

Author, Year	No. Pts	Study	Approach	Rectal Bleeding	Haematuria	Infection	Acute Urinary Retention	Pain	Key Findings
Mian, 2024 [39]	718	RCT, ProBE-PC trial	TR—351 TP—367	Comparable	Comparable	TR—2.6% TR—2.7%	TR—<2% TP—5–11.1%	NA	Non-infectious complications were comparable; sepsis rate was 0% for both approaches.
Hu, 2024 [38]	658	RCT, prevention study	TR—330 TP—328	TR—0.4% TP—0%	NA	TR—1.4% TP—0%	TR—1.1% TP—0.3%	TR—2.3% TP—2.1%	TP group reported more pain and discomfort, but was small and usually resolved within 1 week.
Ploussard, 2024 [22]	250	PERFECT trial (CCAFU-PR1)	TR—128 TP—122	No significant difference	NA	Comparable	TR—4 patients TP—2 patients	No difference	Pain, quality of life urinary and sexual functions were comparable. Sepsis in TR group (0.8%).
Berquin, 2023 [36]	128	Comparative–observational	TR—61 TP—67	TR—3% TP—8.2%	TR—32.8% TP—46.3%	TR—3.3% TP—0%	TR—0% TP—1.5%	Similar	No difference in pain and IPSS among groups; haematuria at 24 h was higher with the TP approach.
Ortner, 2021 [40]		Comparative		TR—36.8% TP—<10%	TR—65.8% TP < 10%	TR—3.6% TP—<0.5%	<2%	43%	Infectious complications are nullified in TP group.
Chen, 2021 [12]	212	Prospective	TR—86 TP—127	NA	TR—1.7% TP—0.9%	TR—2.2% TP—0.9%	TR—4.5% TP—3.8%	TR—0 TP—0.9%	Overall complication rates were 11.2% for TR vs. 6.1% for TP. Non-septic UTIs were lower in TP group.
Berry, 2020 [41]	73,630	Comparative	TR—59,907 TP—13,723	NA	TR—0.66% TP—0.71%	NA	TR—0.95% TP—1.93%	NA	TP biopsy had a lower risk of readmission for sepsis but a higher risk of readmission for urinary retention than TR biopsy.
Lo, 2019 [14]	200	Retrospective, comparative	TR—100 TP—100	TR—1% TP—0%	TR—0 TP—0	TR—4% TP—0	TR—1% TP—3%	NA	Four percent in TR group had fever and required at least 1-week admission for intravenous antibiotic administration.
Xue, 2017 [15]	4230	Meta-analysis	4230	TR—10.2%. TP—1.5%.	TR—20.6%. TP—17.6%.	NA	TR—3.8% TP—2.4%	NA	Overall complications were comparable.
Guo, 2015 [16]	399	Prospective	TR—166 TP—173	TR—8.7% TP—0%	TR—23% TP—19.8%	Sepsis—0% for TP vs. 0.6% TR	NA	TR—13% TP—35%	Higher rates of mild rectal bleeding and mild pain in TP group.

TP—transperineal; TR—transrectal; NA—not available.

## Abbreviations

The following abbreviations are used in this manuscript: PBx—prostate biopsy; TRUS—transrectal ultrasound; OR–odds ratio; CI—confidence interval; TP—transperineal; TR—transrectal; CDR—cancer detection rate; NA—not available; PCa—prostate cancer; csPCa—clinically significant prostate cancer; and mpMRI—multiparametric magnetic resonance imaging.
